# Effects of duration of stay in temperate area on thermoregulatory responses to passive heat exposure in tropical south-east Asian males residing in Japan

**DOI:** 10.1186/1880-6805-31-25

**Published:** 2012-09-13

**Authors:** Titis Wijayanto, Sayo Toramoto, Hitoshi Wakabayashi, Yutaka Tochihara

**Affiliations:** 1 Department of Human Science, Faculty of Design, Kyushu University, 4-9-1, Shiobaru, Minami-ku, Fukuoka, 815-8540, Japan; 2 Education Center, Faculty of Engineering, Chiba Institute of Technology, Chiba, Japan

**Keywords:** Thermoregulatory responses, Heat acclimatization, Decay of acclimatization, Heat tolerance

## Abstract

**Background:**

In this study, we investigated the effects of duration of stay in a temperate area on the thermoregulatory responses to passive heat exposure of residents from tropical areas, particularly to clarify whether they would lose their heat tolerance during passive heat exposure through residence in a temperate country, Japan.

**Methods:**

We enrolled 12 males (mean ± SE age 25.7 ± 1.3 years) from south-east Asian countries who had resided in Japan for a mean of 24.5 ± 5.04 months, and 12 Japanese males (age 24.1 ± 0.9 years). Passive heat exposure was induced through leg immersion in hot water (42°C) for 60 minutes under conditions of 28°C air temperature and 50% relative humidity.

**Results:**

Compared with the Japanese group, the tropical group displayed a higher pre-exposure rectal temperature (*P* < 0.01) and a smaller increase in rectal temperature during 60 minutes of leg immersion (*P* = 0.03). Additionally, the tropical group showed a tendency towards a lower total sweat rate (*P* = 0.06) and lower local sweat rate on the forehead (*P* = 0.07). The tropical group also had a significantly longer sweating onset time on the upper back (*P* = 0.04) compared with the Japanese groups. The tropical group who stayed in Japan for > 23 months sweated earlier on the forehead and upper back than those who stayed in Japan < 11 months (*P* < 0.01 and *P* = 0.03 for the forehead and upper back, respectively). There was a positive correlation between duration of stay in Japan and total sweat rate (*r* = 0.58, *P* <0.05), and negative correlations between duration of stay and sweating onset time on the forehead (*r* = −0.73, *P* = 0.01) and on the upper back (*r* = −0.66, *P* = 0.02). Other physiological indices measured in this study did not show any difference between the subjects in the tropical group who had lived in Japan for a shorter time and those who had lived there for a longer time.

**Conclusions:**

We conclude that the nature of heat acclimatization of the sweating responses to passive heat exposure that are acquired from long-term heat acclimatization is decayed by a stay in a temperate area, as shown by the subjects in our tropical group. We did not find any evidence of a decay in the other physiological indices, indicating that heat tolerance acquired from long-term heat acclimatization is not completely diminished through residence in a temperate area for less than 4 years, although some aspects of this heat tolerance may be decayed.

## Background

A number of studies on heat acclimatization, in terms of physiological and behavioral adaptation, have been reported over the past half century. Residents from tropical areas, who were naturally acclimatized to a hot environment, were reported to have better tolerance to any given heat exposure than residents of temperate areas. In a series of studies on heat acclimatization in tropical natives from Malaysia and temperate natives from Japan [1-3], the Malaysians appeared to display higher rectal temperature under pre-exposure conditions, with smaller increases of rectal temperature during passive heat exposure [3] and during sub-maximal exercise in heat [2]. The tropical native Malaysians also exhibited lower local sweat rate with a longer sweating onset time during heat exposure compared with the Japanese [3]. The aforementioned thermoregulatory responses in tropical natives, including higher core temperature at rest condition [4-7], sweat rate suppression [8,9], a smaller number of activated sweat glands [3,10] and higher skin temperature for enhancing dry heat loss [7,11], have been suggested to be the result of long-term heat acclimatization to physiological functions, and indicate a superior heat tolerance in people from tropical areas [6,9].

It is well established that, in contrast to long-term acclimatization, short-term acclimation enhances heat tolerance by reducing resting and exercise heart rate, lowering resting core temperature and threshold for the onset of sweating [12,13], and inducing higher sweat rate [14-16]. Lind [17] suggested that heat tolerance gained from short-term acclimation might be retained for 2 weeks after the last day of exposure, and then disappears rapidly if it is not maintained by repeated heat exposure. It was reported that heat tolerance disappeared at around 6 to 21 days post-acclimation [15,18] and that every 2 days without working resulted in decay of acclimation [19]. The percentage decay of acclimation is greater for sweat rate and heart rate than for core temperature after ceasing acclimation [15,18,20]. Although studies on the decay of short-term acclimation are well documented, study on the decay of long-term heat acclimatization are relatively rare.

In the late 1970s, Hori *et al*. [21] conducted a study comparing the sweating reaction of migrants from Okinawa (a sub-tropical area) who had lived on the Japanese mainland for less than 3 years with those who had lived on the mainland for more than 10 years. The Japanese from Okinawa were considerably to have more adapted to hot environment since they were born compared with the mainland Japanese; the Japanese from Okinawa were reported to have more advanced heat acclimatization than Japanese in the mainland, even though the Okinawans had lived on the main island of Japan for less than 3 years [22]. In comparison between the migrants from Okinawa who had stayed for less than 3 years and those who had stayed for more than 10 years, the physiological responses of sub-tropical natives from Okinawa decayed after long-term residence in the temperate area [21]. However, even though Okinawa is located in a sub-tropical region and the summer is longer than the winter, there is seasonal variation in thermoregulatory responses to heat in the residents from Okinawa [23]. We suggest therefore that sub tropical natives from Okinawa cannot be considered to have a similar level of acclimatization as those of tropical natives. Saat *et al*. [24] reported that Malaysians who stayed in Japan for more than 27 months had a tendency to have a shorter sweating onset time than those who stayed in Japan for less than 15 months. Additionally, Lee *et al*. [25] found that the sweating onset time was shorter and the sweat volume was greater in Malaysians with a longer duration of stay in Japan for 2 to 72 months, indicating a gradual decay of heat acclimatization of tropical natives residing in temperate area. The aforementioned reports seem to answer the historical debate regarding the importance of genotype [26,27] or phenotype [8,28-30] in heat adaptation; that is, heat acclimatization in tropical natives is a reflection of their physiological adjustment to environmental factors rather than involvement of genetic factors.

A recent study by Lee *et al*. [31] reported another study on the decay of heat acclimatization in tropical natives. Despite their prolonged residence in temperate climate for up to 61 months, these subjects did not show any evidence of decay of heat acclimatization as measured by cutaneous thermal sensitivity. Lee *et al*. [31] considered that this decay in cutaneous thermal sensitivity might occur after the adjustment of sudomotor or vascular activity. In heat acclimation studies [15,32], the decay was greater for heart rate and sweat loss than for rectal temperature, and the heat tolerance gained from short-term heat acclimation did not completely disappear after ceasing acclimation. In addition, although some studies have shown evidence of changes in sweating response was seen after a prolonged stay in a temperate area [24,25], other studies have reported no indication of reduction in cutaneous thermal sensitivity [31]. It seems therefore that there might be stages in the decay of heat acclimatization in tropical natives, depending on the time they spend residing in temperate area, just as there are stages for development and decay in short-term heat-acclimation studies.

Most of the aforementioned studies on the decay of heat acclimatization in tropical natives are based on the local sweat response activated by acetylcholine applied iontophoretically [24,25] or on the cutaneous thermal threshold [31]. Except the study investigating decay of heat tolerance during heat exposure in sub tropical natives from Okinawa [21], there has been less research directly investigates whether heat tolerance during exposure in long-term acclimatized tropical natives is affected by their prolonged residences to temperate area. The present study seeks to investigate the thermoregulatory responses to passive heat exposure in the tropical group who were born and raised in the tropics but moved to live in a temperate area. This study particularly clarified whether the tropical group will lose their heat tolerance during passive heat exposure through residence in a temperate country. We hypothesized that the decay of heat acclimatization in the tropical group might not completely disappear, but would partially decay through prolonged residencies in a temperate climate, indicating stages of decay of heat acclimatization in tropical natives after residing in temperate areas.

## Methods

### Ethics approval

All experimental protocols were approved by the institutional review board of Kyushu University. The purpose of the study and the procedures were explained to the subjects before they provided consent.

### Subjects

Twelve male students from south-east Asian countries (tropical group (TR): five Indonesians, four Vietnamese, one Thai, one Filipino, and one Malaysian) who had been residing in Fukuoka, Japan for 24.5 ± 5.04 months (range 4 to 47 months) before the experiment, were enrolled in the study. The control group was comprised of 12 Japanese male students who were born and raised in Japan (Japanese group, JP). The TR subjects were born and raised in the tropical countries, defined as countries with hot and humid weather and with two seasons (dry and rainy).

The morphological characteristics for the TR group were (mean ± SE) age 25.67 ± 1.28 years (range 19 to 33 years), height 173.23 ± 1.53 cm (range 164.5 to 183.0 cm), body mass 63.55 ± 1.34 kg (range 55.73 to 69.97 kg), total body surface area (BSA) 1.80 ± 0.03 m^2^ (range 1.67 to 1.95 m^2^), BSA/body mass 0.028 ± 0.0003 m^2^/kg (range 0.027 to 0.029 m^2^/kg), and percentage body fat (BF) 20.03 ± 1.53% (range 13.1 to 31.8%). The characteristics for the JP group were age 24.08 ± 0.91 years (range 22 to 33 years), height 169.93 ± 1.64 cm (range 162 to 177.6 cm), body mass 59.70 ± 2.45 kg (range 46.75 to 72.92 kg), total BSA 1.72 ± 0.04 m^2^ (range 1.50 to 1.91 m^2^), BSA/body mass 0.029 ± 0.0006 m^2^/kg (range 0.026 to 0.032 m^2^/kg), and percentage BF 19.96 ± 1.25 (range 14.8 to max 25.9%). There were no significant differences in morphological characteristics between TR and JP group.

Subjects in both groups were all undergraduate or graduate school students with a similar physical activity level. Most of the subjects were not currently engaged in any endurance sports activity, either personally or as a group activity. No major differences in dietary habits were apparent between the two groups, and none of the subjects was vegetarian. Subjects in the TR group did not report any significant change in the composition of their diet after arriving in Japan. All the subjects in the TR group reported no residency in any temperate country for more than one year before coming to Japan.

Based on the length of their stay in Japan, the TR group was divided into two groups: TR-S (n = 5) for those who had been living in Japan for 4 to 12 months, and TR-L (n = 7) for those who had been living in Japan for 23 to 47 months. There are no significant differences in the morphological characteristics between TR-S and TR-L (Table 1). For all the subjects in the TR-S group, they had experienced winter for the first time during their stay in Japan.

**Table 1:** **Morphological characteristics of the tropical group (TR) and its sub-groups (short stay (TR-S), and long stay (TR-L)) and the Japanese group (JP) **^**1 **^

	**TR-S,**^**2**^**n = 5**	**TR-L,**^**3**^**n = 7**	**TR, n = 12**	**JP, n = 12**
Age, years	23.40 ± 1.86	27.29 ± 1.57	25.67 ± 1.28	24.08 ± 0.91
Body height, cm	173.52 ± 2.35	173.01 ± 2.18	173.23 ± 1.53	169.93 ± 1.64
Body mass, kg	63.44 ± 1.88	63.63 ± 1.99	63.55 ± 1.34	59.70 ± 2.45
BSA^4^, m^2^	1.80 ± 0.03	1.80 ± 0.04	1.80 ± 0.03	1.72 ± 0.04
BSA/body mass, m^2^/kg	0.03 ± 0.0004	0.03 ± 0.0004	0.03 ± 0.0003	0.03 ± 0.0006
Body fat, %	18.86 ± 1.81	21.43 ± 2.11	20.03 ± 1.53	19.96 ± 1.25

^1^Data are presented as mean ± standard error.

^2^TR-S, Tropical subjects who had stayed in Japan for < 11 months before the experiment.

^3^TR-L, Tropical subjects who had stayed in Japan for > 23 months before the experiment.

^4^BSA, body surface area.

### Experimental procedures

Subjects wore only shorts during the experiment and they maintained a sitting position on a chair in an environmental chamber maintained at an air temperature of 28°C and 50% RH for more than 40 minutes to allow attachment of sensors. A 10-minute stabilization period was then allowed for baseline measurement, followed by 60 minutes of passive heating in the same environmental chamber, which was induced through immersion of the lower legs to the knees in water at 42°C. Leg immersion in hot water has been experimentally used previously to investigate the heating of subjects in order to raise body temperature and enhance sweating [3,6,9]. To minimize any circadian rhythm effect on the physiological responses, each experimental session was started at 13.00 for all subjects after they were given an initial rest period of 2 hours, during which they lay in the supine position in a chamber maintained at an air temperature of 28°C and 50% RH. The same experimental protocol was applied to both the TR and JP subjects. The experiment was performed in winter for both TR and JP groups.

### Measurements

Rectal temperature (T_re_) and skin temperatures were monitored every 2 seconds with thermistor probes (LT-8A; Gram Corporation, Saitama, Japan). T_re_ was monitored with a thermistor probe inserted 130 mm beyond the anal sphincter throughout the test, and skin temperatures were monitored at 10 body sites: forehead, upper back, chest, abdomen, upper arm, forearm, hand, thigh, calf, and foot. Mean skin temperature (${\bar{T}}_{\mathrm{SK}}$) was calculated using the modified Hardy and DuBois’ equation:

${\bar{T}}_{\mathrm{sk}}$= 0.07T_forehead_ + 0.35(T_chest_ + T_abdomen_ + T_upperback_)/3 + 0.14(T_upperarm_ + T_forearm_)/2 + 0.05T_hand_ + 0.19T_thigh_ +  0.13T_calf_ + 0.07T_foot_.

Heart rate (HR) was measured every second using an HR monitor (RS400; Polar Electro Oy, Kempele, Finland) placed around the subject’s chest. Forearm blood flow (FBF; expressed as ml/100 ml) was measured by venous occlusion plethysmography with a strain gauge containing mercury in an elastic tube placed around one-third of the upper right forearm positioned above the heart level. The hand was eliminated from the circulation by a wrist cuff inflated to 200 mmHg, and venous return from the forearm was occluded with an upper-arm cuff inflated to 50 mmHg. FBF was measured three times at 5 minutes before starting leg immersion as baseline, and every 10 minutes after the commencement of leg immersion, and the three readings were averaged.

Systolic blood pressure (SBP) and diastolic blood pressure (DBP) were measured in the right upper arm at the heart level using automatic tonometer (HEM-737, OMRON, Japan) at 5 minutes before leg immersion and every 10 minutes thereafter. Mean arterial blood pressure (MAP) was calculated as

MAP = DBP + (SBP − DBP)/3.

Forearm vascular conductance (FVC) was defined as the ratio of respective FBF to MAP. The relative values of FBF and FVC were calculated as the percentage change in FBF and FVC at the end point of the leg immersion session relative to the baseline value.

Total body sweat rate (${\dot{M}}_{\text{sw}}$) was calculated from nude body mass measured before and after leg immersion using a calibrated scale (Mettler ID2 MultiRange; Mettler-Toledo GmbH, August Sauter, Germany) with a minimum calibration of 1 g. Subjects were instructed to towel-dry themselves thoroughly before body mass measurement. Actual total body mass loss during leg immersion was corrected for insensible body mass loss, and then divided by total leg immersion time (60 minutes) and BSA. Insensible body mass loss was estimated using the differences in body mass before and after the initial 2 hour rest in the chamber maintained at an air temperature of 28°C and 50% RH. Local sweat rate ${\dot{m}}_{\mathrm{sw}}$ on the forehead, back (left scapula), and left forearm (ventral, mid-anterior) were measured continuously using a ventilating capsule method hydrometer (Atmo Chart SS-100II; Kands Co. Ltd, Kariya, Japan). A cylindrical sweat capsule with a diameter of 11.2 mm, which covered a skin area of 1 cm^2^, was mounted on each body region measured in this study, and was ventilated with dry air at a rate of 0.3 L/min. Sweating onset time (OT) was determined as the time until a prompt increase of ${\dot{m}}_{\mathrm{sw}}$ after the commencement of leg immersion.

### Data analysis

Data and figures are presented as mean ± standard error (SE). Values after 60 minutes of leg immersion were calculated from the mean readings during the last 5 minutes (from 55 to 60 minutes). Statistical analysis was performed using SPSS software (version 15 for Windows; SPSS Inc., Chicago, IL, USA). Data from time-course measurements were evaluated using repeated measures ANOVA (time × group) with Tukey’s *post hoc* test to identify the overall difference between TR and JP. We additionally treated the data of local sweat rate on the forehead, upper back, and forearm using repeated measures ANOVA (time × region) to test the local sweat rate distribution in both group. In cases where no significant interaction was shown by two-way ANOVA, the unpaired Student’s *t*-test was performed to test group differences between TR and JP subjects for each point of time. Total sweat rate and sweating OT were compared using the unpaired Student’s *t*-test between the TR and JP group, and between the TR-S group and TR-L group. The relationships between the duration of stay in Japan and physiological indices measured during leg immersion in the TR group were examined using Pearson’s correlation coefficient analysis. *P* < 0.05 was considered significant with *P* < 0.1 indicating a tendency toward a difference.

## Results

### Thermoregulatory responses to heat in TR and JP

**Figure 1:**
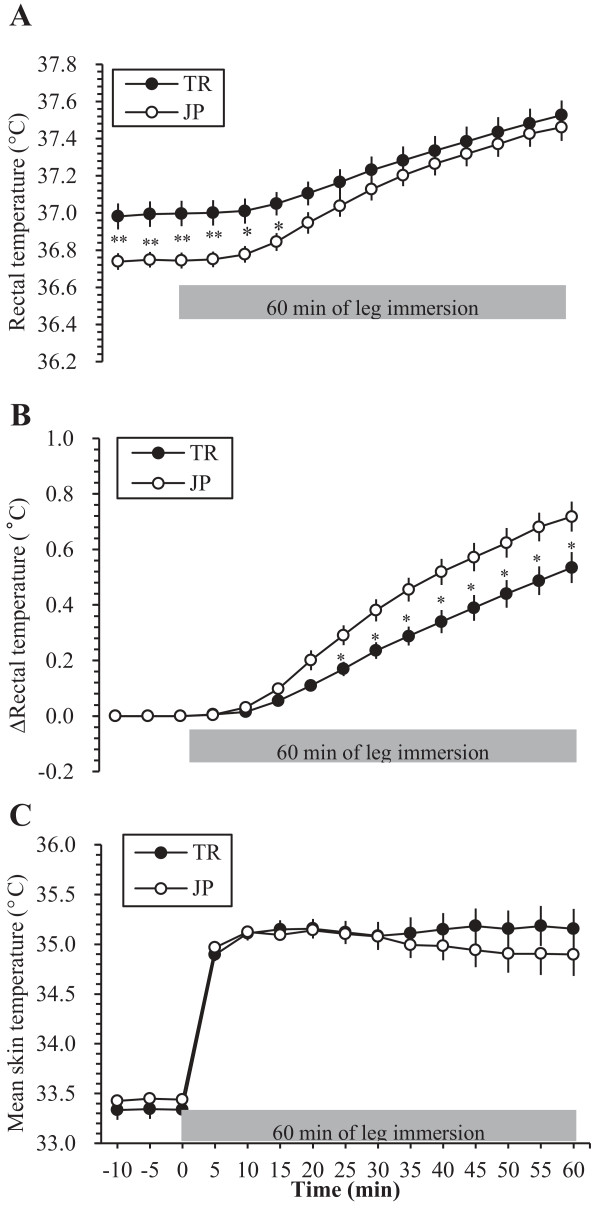
**Change in temperature during 10 minutes of stabilization and 60 minutes of leg immersion in the tropical (TR) and Japanese (JP) groups.** (**A**) Rectal temperature; (**B**) change in rectal temperature; (**C**) mean skin temperature. ^*^P < 0.05 between TR and JP ; ^**^P < 0.01 between TR and JP.

Figure 1 illustrates the time course and rise in T_re_, ΔT_re_, and ${\bar{T}}_{\mathrm{sk}}$ during 10 minutes of stabilization and 60 minutes of leg immersion in the TR and JP groups. The initial value of T_re_ before starting leg immersion was significantly higher in the TR than in the JP group (37.00 ± 0.06°C and 36.74 ± 0.04°C, respectively; *P* < 0.01), and this continued until 15 minutes of leg immersion (*P* < 0.05). At the end of 60 minutes of leg immersion T_re_ reached 37.53 ± 0.08°C for TR and 37.46 ± 0.07°C for JP, which was not significantly different between the two groups (*P* = 0.55). The reason for this lack of significant difference in T_re_ was due to a significantly greater ΔT_re_ in the JP group (*P* = 0.05) starting from 20 minutes after the commencement of leg immersion (Figure 1B). There were no significant differences in ${\bar{T}}_{\mathrm{sk}}$ between the TR and JP groups before (33.33 ± 0.10°C and 33.43 ± 0.06°C, respectively; *P* = 0.43), and after 60 minutes of leg immersion (35.15 ± 0.13°C and 34.90 ± 0.22°C, respectively; *P* = 0.39).

There was no significant difference in HR between the TR and JP groups before (79 ± 3 beats/min and 73 ± 3 beats/min, respectively; *P* = 0.21) or after 60 minutes of leg immersion (101 ± 3 beats/min and 96 ± 5 beats/min, respectively; *P* = 0.37). ΔHR after 60 minutes of leg immersion did not differ between the TR and JP groups (22 ± 3 beats/min and 22 ± 3 beats/min, respectively; *P* = 0.93).

FBF at the baseline tended to be higher in the TR (3.42 ± 0.32 ml/100 ml) than in the JP group (2.61 ± 0.31 ml/100 ml) but did not reach statistical significance (*P* = 0.07). At the end point of leg immersion, FBF did not significantly differ between TR and JP (6.47 ± 0.61 ml/100 ml and 7.14 ± 0.58 ml/100 ml, respectively; *P* = 0.43). The relative change in FBF after 60 minutes of leg immersion was higher in the JP than in the TR group (193 ± 24% and 92 ± 13%, respectively; *P* = 0.04).

There were no significant differences in MAP between the TR and JP groups at baseline (81 ± 2 mmHg and 85 ± 2 mmHg, respectively; *P* = 0.18) or after 60 minutes of leg immersion (81 ± 3 mmHg and 87 ± 3 mmHg, respectively; *P* = 0.11).

FCV was significantly higher in the TR (0.04 ± 0.01 ml/100 ml/mmHg) than in the JP group at baseline (0.03 ± 0.01 ml/100 ml/mmHg; *P* = 0.04), but not at the end point of leg immersion (0.08 ± 0.01 ml/100 ml/mmHg and 0.08 ± 0.01 ml/100 ml/mmHg, respectively; *P* = 0.84). The relative change in FVC after 60 minutes of leg immersion was significantly higher in the JP than in the TR group (185 ± 26% and 94 ± 15%, respectively; *P* = 0.006).

**Figure 2:**
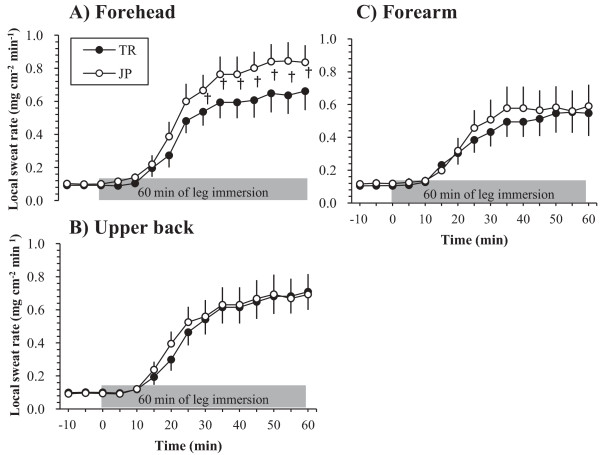
**Local sweat rate during 10 minutes of stabilization and 60 minutes of leg immersion in the tropical (TR) and Japanese (JP) groups.** (**A**) Forehead; (**B**) Upper back; (**C**) Forearm. † *P* < 0.1 between TR and JP.

${\dot{M}}_{\text{sw}}$ during 60 minutes of leg immersion showed a tendency to be lower in TR than in JP (71.05 ± 4.93 g/m^2^/h and 88.33 ± 6.99 g/m^2^/h; *P* > 0.05). ${\dot{m}}_{\mathrm{sw}}$ on the forehead during 60 minutes of leg immersion tended to be lower in the TR than in the JP group (*P* = 0.07) (Figure 2A). Meanwhile, ${\dot{m}}_{\mathrm{sw}}$ on the forearm and upper back during 60 minutes of leg immersion showed no differences between the TR and the JP group (*P* > 0.05) (Figure 2B,C).

Sweating OT on the upper back after the start of leg immersion was longer in the TR group than in the JP group (*P* = 0.04), whereas there were no differences between the two groups in OT for the forehead (*P* = 0.16) or for the forearm (*P* = 0.42).

### Relationship between physiological responses and duration of stay in Japan

Table 2 summarizes the physiological responses of subjects during 60 minutes of leg immersion in all groups. There were no significant differences in T_re_ and, ${\bar{T}}_{\mathrm{sk}}$ between the TR-S and TR-L sub-groups, either at baseline or after 60 minutes of leg immersion. There was no difference in ΔT_re_ between TR-S and TR-L, but there was a tendency towards a smaller ΔT_re_ in TR-L and TR-S compared with ΔT_re_ in JP, although this was not significant (*P* = 0.07 for TR-L and *P* = 0.08 and TR-S, respectively). Other physiological responses such as HR, ΔHR, FBF, FCV and the relative change in FBF and FCV after 60 minutes of leg immersion were not significantly different between TR-S and TR-L. However, the relative changes of FCV were significantly lower in TR-S and TR-L compared with JP (*P* = 0.04 for TR-S and *P* = 0.03 TR-L).

**Table 2:** **Physiological responses to 60 minutes of leg immersion of subjects in the tropical Asian group (TR, TR-S, TR-L) and the Japanese group (JP)**^1^

**Variable**	**TR-S,**^**2**^**n = 5**	**TR-L, n = 7**	**TR. n = 12**	**JP, n = 12**
Initial T_re_,°C	37.02 ± 0.14^c^	36.98 ± 0.06^c^	37.00 ± 0.06^d^	36.74 ± 0.04
Final T_re_,°C	37.62 ± 0.12	37.49 ± 0.08	37.53 ± 0.08	37.46 ± 0.07
ΔT_re_,°C	+0.60 ± 0.07^e^	+0.51 ± 0.07^e^	+0.53 ± 0.06^c^	+0.72 ± 0.05
Initial ${\bar{T}}_{\mathrm{sk}}$,°C	33.29 ± 0.12	33.37 ± 0.15	33.33 ± 0.10	33.43 ± 0.06
Final ${\bar{T}}_{\mathrm{sk}}$,°C	35.09 ± 0.18	35.20 ± 0.33	35.15 ± 0.13	34.90 ± 0.22
HR, beats/min	76 ± 3	81 ± 4	79 ± 3	73 ± 3
ΔHR, beats/min	27 ± 3	20 ± 4	22 ± 3	22 ± 3
Baseline FBF, ml/100 ml	2.96 ± 0.25^e^	3.75 ± 0.49^e^	3.42 ± .32^e^	2.61 ± 0.29
Final FBF, ml/100 ml	5.92 ± 0.53	6.87 ± 0.92	6.47 ± 0.43	7.14 ± 0.58
Relative change in FBF, %	99 ± 16^c^	88 ± 20^c^	92 ± 13^c^	193 ± 24
Baseline FVC, ml/100 ml/mmHg	0.04 ± 0.01^e^	0.047 ± 0.01^e^	0.042 ± 0.00^c^	0.03 ± 0.03
Final FVC, ml/100 ml/mmHg	0.07 ± 0.01	0.09 ± 0.01	0.08 ± 0.01	0.08 ± 0.01
Relative change in FVC, %	97 ± 16^c^	93 ± 24^c^	94 ± 15^d^	185 ± 26
${\dot{M}}_{\text{sw}}$, g/m^2^/h	63.1 ± 8.10^e^	79.0 ± 6.1	71.64 ± 4.93^e^	88.33 ± 6.99
OT on the forehead, minutes	17.9 ± 0.8^f^,^c^	11.2 ± 17.9	14.5 ± 1.37	11.7 ± 1.4
OT on the upper back, minutes	17.6 ± 0.8^f^,^3^	11.3 ± 0.8	14.4 ± 1.45^3^	10.3 ± 1.2
OT on the forearm, minutes	13.0 ± 2.0^g^	8.8 ± 1.4	10.9 ± 1.33	12.5 ± 1.4

^a^Data are presented as mean ± standard error.

^b^Abbreviations: T_re_, rectal temperature; ΔT_re_, rise in rectal temperature after 60 minutes of leg immersion; ${\bar{T}}_{\mathrm{sk}}$, mean skin temperatures; FBF, forearm blood flow; FVC, forearm vascular conductance; HR, heart rate; ΔHR, change in heart rate after 60 minutes of leg immersion; ${\dot{M}}_{\text{sw}}$, total sweat rate; OT, sweating onset time; TR-L, Tropical subjects who had stayed in Japan for > 23 months before the experiment; TR-S, Tropical subjects who had stayed in Japan for < 11 months before the experiment.

^c^*P* < 0.05 compared with the JP group.

^d^*P* < 0.01 compared with the JP group.

^e^*P* < 0.1 compared with the JP group.

^f^*P* < 0.05 between TR-S and TR-L.

^g^*P* < 0.1 between TR-S and TR-L.

The ${\dot{M}}_{\text{sw}}$ during 60 minutes of leg immersion looks smaller in TR-S group than in TR-L group (Table 2) however, there was no significant difference between the TR-S and TR-L groups (*P* = 0.31). ${\dot{M}}_{\text{sw}}$ tended to be lower in the TR-S than in the JP group (*P* = 0.07), whereas there was no significant difference between the TR-L and the JP groups (*P* = 0.23). OT for the forehead and upper back was significantly longer in the TR-S than the TR-L group (*P* < 0.01 for the forehead and *P* = 0.03 for the upper back), whereas OT for the forearm tended to be longer in TR-S than in TR-L (*P* = 0.07). The TR-S group had a significantly longer OT on the forehead and upper back compared with the JP group (*P* = 0.01 for the forehead and *P* < 0.001 for the upper back), whereas there were no differences for the comparison between the TR-L and JP groups.

**Figure 3:**
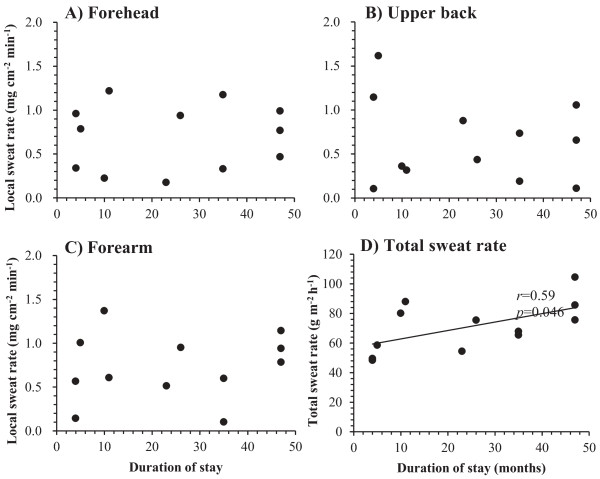
**Relationship between duration of stay in Japan and sweat rate. (A-C) Relationship between duration of stay in Japan and local sweat rate after 60 minutes of leg immersion on:** (**A**) Forehead; (**B**) Upper back; (**C**) Forearm. (**D**) **Relationship between duration of stay in Japan and total sweat rate after 60 minutes of leg immersion.** * *P* < 0.05.

**Figure 4:**
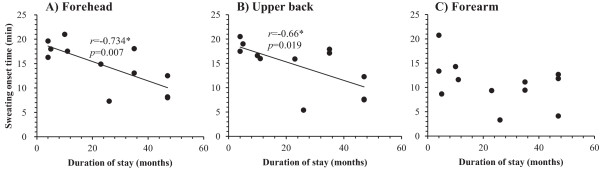
**Relationship between duration of stay in Japan and sweating onset time.** (**A**) Forehead; **B**) Upper back; **C**) Forearm.^*^P < 0.05.

For the TR group, significant relationships were found between duration of stay in Japan and the ${\dot{M}}_{\text{sw}}$ (r = 0.59, *P* < 0.05) (Figure 3D), and ${\dot{M}}_{\text{sw}}$ increased as the duration of stay in Japan increased. No statistical correlations between duration of stay and ${\dot{m}}_{\mathrm{sw}}$ were seen for any of the measured body regions. OT readings on the forehead (r = −0.73, *P* < 0.01; Figure 4A) and upper back (r = −0.66, *P* = 0.02; Figure 4B) were negatively correlated with duration of stay in Japan, and they decreased as the duration of stay in Japan increased (from 4 to 47 months). There were no significant relationships seen between duration of stay and other physiological responses during 60 minutes of leg immersion (*P* > 0.05).

## Discussion

It is generally agreed that heat acclimatization is mainly influenced by environmental factors [28-30], although some authors believe that genetic factors are more important [26,27]. Heat tolerance gained from long-term heat adaptation was reported to be decayed after prolonged residence in temperate areas [21,22,24,25]. In the present study, we observed the changes in sweating reaction, as indicated by a shortened sweating OT and an increased in total sweat rate, in subjects who had immigrated from their native countries in tropical regions to the temperate country of Japan, and had stayed for up to 47 months in this temperate area.

The subjects who stayed in Japan for longer than 23 months (TR-L) sweated earlier on the forehead and upper back than those who stayed in Japan for less than 11 months (TR-S) (Table 2). The sweating OT of the TR group became shorter as the duration of stay in Japan increased (Figure 4). These findings corroborate the study of Saat *et al*. [24] and Lee *et al*. [25], who reported changes in sweating reaction as the duration of stay in Japan increased. Prolonged residence in temperate areas by tropical natives modified the sweating mechanism in the direction typical for temperate natives, characterized by an earlier onset of sweating [25]. In contrast to a previous study, which found that Okinawa residents who stayed in the Japanese mainland for more than 10 years had greater body weight loss and greater peak local sweating than those who stayed for shorter periods [21], we did not find any significant differences in total sweat rate between the TR group with longer stay and those with shorter stay (Table 2). However, the total sweat rate of those TR subjects who had lived in Japan for more than 23 months but less than 47 months did not differ inform those of the JP group. In addition, total sweat rate increased linearly with the duration of stay in Japan (Figure 3D).

We did not find any evidence for decay of heat acclimatization for the other physiological indices measured in this study. These other physiological responses to heat were in agreement with the results of other studies, including smaller increases in rectal temperature [1,3,6], lower local sweat rate on the forehead [3], lesser degrees of sweating [9,10,25,27,33], a delay in sweating OT [9,34,35] and lower forearm blood flow [3,27-29] in the tropical group compared with the Japanese group, reinforcing the current state of heat acclimatization in tropical natives that has been reported in earlier studies. These physiological indices, except for total sweat rate and sweating OT, did not change as the period of stay increased. Hori *et al*. [22] reported that Japanese subjects from Okinawa reflected more advanced heat acclimatization than mainland Japanese subjects, even though the former had migrated to the main island of Japan less than 3 years previously. In a short-term heat-acclimation study, the percentage decay of acclimation was greater for sweat rate and HR than for core temperature after ceasing acclimation [15,18,20]. Weller *et al*. [20] suggested that decay in HR and sweat loss were greater than that in rectal temperature in their study because the cardiovascular adaptations are the first to adapt during heat acclimation and thus are also the first to decay, whereas the adjustments in sweat rate are the last to adapt during heat acclimation [32]. In the present study, decay in HR and T_re_ were not apparent, whereas a decay in sweating responses was evident. These results suggest that the smaller rise in T_re_ and other physiological indices, except for sweating responses, in tropical natives were not altered by a prolonged stay in a temperate area.

However, several factors that might result in the decay of acclimatization in this study need to be taken in to account. The first factor is the length of stay in the temperate area; in this study, the mean duration of stay of the TR group in Japan less than 4 years, which might not be sufficiently long to produce significant changes in thermoregulatory responses to heat. As reported by Hori *et al*. [21], changes in sweating response can be clearly seen in subjects after migrating and staying in a temperate area for more than 10 years. Additionally, Kuno [8] suggested that residence for more than 6 years is necessary to acquire the same capacity as people native to that area. Secondly, after moving to and residing in a temperate area, tropical natives will have experienced different seasons. Thus, seasonal variation in thermoregulatory responses might exist, not only in the temperate natives, who clearly show seasonal variations in physiological responses to heat [36,37], but also in tropical natives during their prolonged residence in temperate areas. Third, other factors such as changes in dietary habits and physical activities are possible underlying factors explaining the decay of acclimatization in the present study. However, in this study, it was difficult to control the third factors during their prolonged stayed in Japan. Hence, the effect of these third factors on the decay of acclimatization might be negligible. Further studies on physiological responses to heat in tropical natives will need to take into account the above factors in order to obtain a comprehensive understanding of decay of acclimatization in tropical natives who are residing in temperate areas.

## Conclusion

There are two salient points of the present study. First, the sweating responses to passive heat exposure of tropical natives who were born and raised in the tropics but had stayed in Japan for up to 47 months decayed during their prolonged stay in this temperate area, signifying a time-dependent characteristic. Second, a smaller increase in rectal temperature in tropical subjects and other physiological indices, except for sweating responses, were not altered by prolonged stay in temperate area. From these findings, we concluded that the nature of heat acclimatization of the sweating response to passive heat exposure that the tropical group has acquired from long-term heat acclimatization started to decay after they moved to a temperate area. No evidence for decay in the other physiological indices, indicated that the heat tolerance the tropical group had acquired from long-term heat acclimatization may be retained, and although it partially decays, it does not completely diminish during a residence of for less than 4 years in a temperate area. The decay in sweating response after having stayed in temperate area seen in the present study indicates that heat in tropical natives reflects physiological adjustments to environmental condition rather than genetic factors.

## Competing interests

The authors declare that they have no competing interests.

## Authors’ contributions

TW contributed to the conception and design of study, data analysis, and interpretation, and wrote the manuscript; HW contributed to the design of the study and the analysis; ST contributed to the data collection; and YT had the overall responsibility for the conception and study design. All authors read and approved the final manuscript.
